# Partial Nephrectomy for a Massive Sporadic Renal Angiomyolipoma: Case Report and Review of the Literature

**DOI:** 10.1155/2016/3420741

**Published:** 2016-12-14

**Authors:** Jacob Albersheim-Carter, Molly Klein, Paari Murugan, Christopher J. Weight

**Affiliations:** University of Minnesota Medical School, Minneapolis, MN, USA

## Abstract

*Introduction. *Angiomyolipomas are the most common benign tumor of the kidney, associated with Tuberous Sclerosis in 20% of cases and arising sporadically in 80% of cases. Renal angiomyolipomas are neoplasms of mesenchymal origin with varying proportions of vasculature, smooth muscle spindle cells, and adipocytes, making management of such neoplasms a challenging endeavor. Possible management options include partial or radical nephrectomy and segmental renal artery embolization.* Case Presentation. *A 61-year-old woman admitted for a large retroperitoneal hemorrhage was discovered to have a giant, sporadic, 3818.3 g, 30.0 × 26.5 × 18.0 cm left perinephric angiomyolipoma. Given her hemodynamic instability upon presentation, she underwent segmental arterial embolization, followed by an open left partial nephrectomy. Ten-month follow-up revealed no noticeable loss of renal function.* Discussion.* Literature review revealed occasional renal angiomyolipomas of comparable size, with all angiomyolipomas larger than this requiring treatment with radical nephrectomy.* Conclusion. *We show that nephron-sparing surgery may be considered in the treatment of even the largest of renal angiomyolipomas.

## 1. Introduction

Angiomyolipomas (AMLs), a type of perivascular epithelioid cell tumor (PEComa), are the most common benign tumor of the kidney, yet are relatively uncommon in the general population, occurring in 0.3%–2.1% of people. Women are more frequently affected than men, with a female-to-male ratio of 11 : 4 [1]. Renal AMLs may arise sporadically or as a consequence of tuberous sclerosis complex (TSC). AMLs arising sporadically have displayed different characteristics compared to AMLs associated with TSC. For example, TSC AMLs have exhibited larger average sizes, more frequent multiple and bilateral lesions, faster growth rate, and earlier presentation compared to sporadic AMLs [2]. In the past, intervention for symptomatic AMLs has included radical nephrectomy or partial nephrectomy, with or without arterial embolization, and embolization alone [3]. We report a clinical case of symptomatic giant sporadic AML in a 61-year-old woman treated with segmental arterial embolization (SAE) followed closely by partial nephrectomy with maintenance of normal renal function.

## 2. Case Presentation

**Figure 1:**
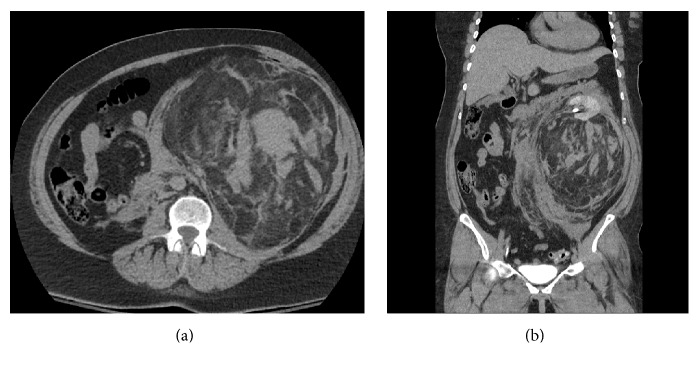
Axial (a) and coronal (b) views of abdominal CT showing massive perinephric AML.

A 61-year-old woman with no history of* TSC1*/*TSC2* mutation or symptoms of TSC presented to the emergency department after a fall from standing level. She subsequently developed hemodynamic instability from a retroperitoneal bleed secondary to hemorrhage of a massive left lower pole perinephric mass containing few small vessels, as well as mixed fatty and soft tissue elements as seen on computerized tomography (Figure 1).

The normal appearing portions of her left kidney were displaced superiorly, but hydronephrosis was not appreciated. After IV fluid resuscitation and stabilization in the emergency room, her care was transferred to the intensive care unit.

She required transfusion of 5 units of packed red blood cells initially, and her hemoglobin continued to drop slowly while in the hospital. Therefore, her care team decided to proceed with SAE of her AML. This would stop the immediate bleeding, but still allow an attempt at a partial nephrectomy.

Four days later, she underwent an open exploration of her left renal AML, with plan for intraoperative evaluation and consideration of a partial versus radical nephrectomy based on the extent of bleeding and inflammation in the retroperitoneal space. Her preoperative hemoglobin was 7.8 g/dL with creatinine of 0.66 mg/dL and eGFR of 74 mL/min/1.7 m^2^. She received four additional units of packed RBCs intraoperatively. Surgical exploration revealed two left renal arteries, including one artery to the AML emanating directly from the aorta, which was ligated and divided, and a second to the main portion of the normal kidney. This artery was clamped for a total of 20 minutes of warm ischemia time. Intraoperative ultrasound was used to identify the transition of normal kidney to kidney tumor. The mass was isolated and excised with successful partial nephrectomy, and the patient left the operating room in stable condition.

**Figure 2:**
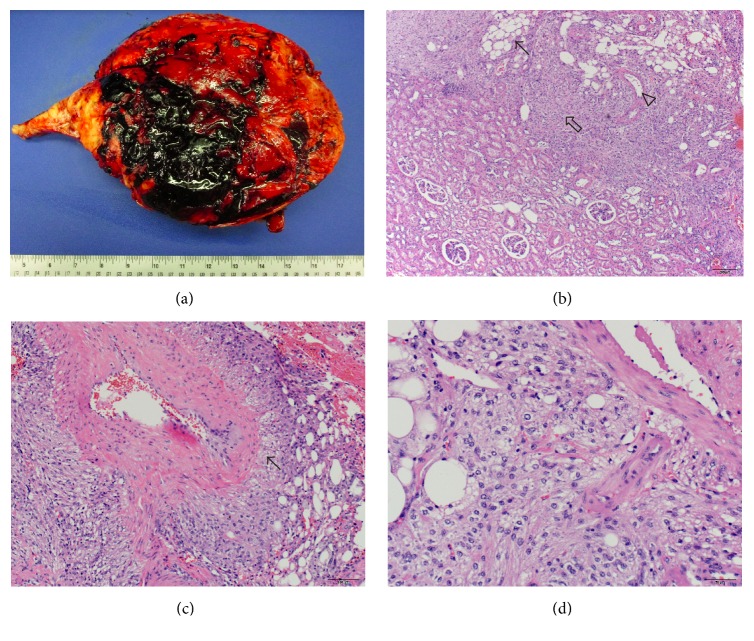
Macroscopic image showing a large circumscribed tumor with extensive hemorrhage (a). Photomicrographs show, arising from the kidney (lower left), an unencapsulated triphasic tumor (upper right) composed of mature adipose tissue (arrow), dystrophic vasculature (arrow head), and smooth muscle (clear arrow) (b). The smooth muscle cells appear to emanate from the wall of an irregular, thick-walled blood vessel (arrow) (c) and are characterized by oval to spindle cells with pale eosinophilic cytoplasm and regular nuclei with small nucleoli (perivascular epithelioid cells) (d).

Pathologic examination revealed a 3818.3 g, 30.0 × 26.5 × 18.0 cm tumor that was grossly well circumscribed and appeared to be attached to the kidney by a stalk. The cut surface revealed a heterogeneous pink-tan to yellow mass with extensive hemorrhage. The microscopic sections showed an unencapsulated, circumscribed triphasic tumor arising from the kidney, composed of prominent, irregular, thick-walled dystrophic blood vessels, interspersed with mature adipose tissue and smooth muscle fascicles, typical of an AML. The smooth muscle cells were oval to spindle with pale eosinophilic cytoplasm, round to oval regular nuclei, and small nucleoli. They appeared to be emanating from the wall of the blood vessels, characteristic of the PEComa family of tumors (Figure 2).

**Figure 3:**
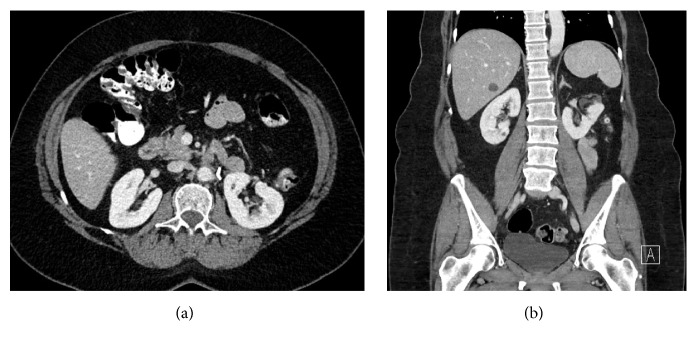
Axial (a) and coronal (b) views of abdominal CT showing recovered left kidney, ten months after partial nephrectomy for removal of a giant AML.

Ten-month postoperative radiological surveillance revealed no evidence of recurrent mass lesion or other postoperative complications (Figure 3). Renal function was virtually unchanged at this time with a creatinine of 0.83 mg/dL and eGFR of 70 mL/min/1.7 m^2^.

## 3. Discussion

The above presentation highlights the challenge of managing symptomatic giant renal AMLs. In the past, asymptomatic renal AMLs smaller than 4 cm in diameter have been followed with imaging studies. Even larger renal AMLs, up to 8 cm in diameter, can be managed without intervention if asymptomatic [4, 5]. For symptomatic patients in whom intervention is indicated, surgical intervention should be considered. Although increased use of cross-sectional imaging has increased the rate of incidental AML findings, up to 15% of renal AMLs still present with hemorrhage, and up to 10% present with hemorrhagic shock [3]. Attempts at minimally invasive nephron-sparing surgery should be made when possible to maximize preservation of renal function and minimize perioperative morbidity, since even the largest of tumors often emanate from a rather small portion of the normal kidney [3, 6, 7]. Historically, surgical intervention has been utilized more frequently than embolization for renal AMLs. However, SAE has proven to be highly efficacious in the management of renal AMLs associated with hemodynamic instability secondary to hemorrhage [8–15]. In our case, SAE was used primarily to control bleeding, thus helping stabilize the patient for attempted partial nephrectomy.

Giant renal AMLs of this magnitude are exceedingly uncommon. Review of literature reveals few cases of giant renal AMLs larger than this, all of which required radical nephrectomy for definitive treatment, with variable outcome [16–21]. Several cases of giant renal AMLs excised using partial nephrectomy have been reported, but to our knowledge, none have been as voluminous as the renal AML in the patient we have presented [22–32]. Exophytic renal AMLs of this size may be easily confused with malignant conditions on CT imaging, such as well-differentiated forms of perirenal liposarcomas. However, sharp defects in the renal parenchyma and the presence of enlarged vessels on CT imaging favor the diagnosis of renal AML. Although possible, very rarely has liposarcoma been found to arise from within the renal parenchyma [33]. Admittedly, giant asymptomatic renal AMLs and giant retroperitoneal liposarcomas both necessitate surgical removal. Therefore, the differentiation of these two conditions with imaging is not imperative, but both diagnoses should be kept in the differential. However, treatment for a massive symptomatic and hemorrhagic renal AML and massive retroperitoneal liposarcoma would be the same, and consideration would still be given for SAE and nephron-sparing surgery.

This case is unique in that the giant AML in this patient was sporadic and not associated with TSC. In patients with TSC who develop AMLs, periodic monitoring of progression of the tumors with ultrasound or MRI is often necessary [2]. More recently, mammalian target of rapamycin (mTOR) inhibitors such as sirolimus have shown promising results in reducing rates of cellular proliferation of AMLs in patients with TSC, as these patients are often found to have upregulation of mTOR in tumor cell lines [34]. Presence of multiple AMLs with larger average sizes in the setting of TSC would likely interfere with the ability to perform nephron-sparing surgery, but as seen in this case, partial nephrectomy should be strongly considered. mTOR inhibitors may have the potential to play a role in making this possible. Consideration should also be given to the increased intraoperative and postoperative complications associated with increasing tumor size [26–31].

## 4. Conclusion

Partial nephrectomy for giant sporadic renal AMLs is possible and, in our opinion, should be considered as an alternative to radical nephrectomy. SAE may also be used to control bleeding preoperatively, regardless of potential tumor shrinkage time.
